# Concerns regarding the safety and efficacy of ES-Cu-Captisol for Menkes disease

**DOI:** 10.1172/JCI201900

**Published:** 2026-02-16

**Authors:** Stephen G. Kaler

**Affiliations:** Department of Pediatrics, Vagelos College of Physicians and Surgeons, Columbia University, New York, New York, USA.

**Keywords:** Clinical Research, Genetics, Neuroscience, Neurological disorders

**To the Editor:** I read with interest the research letter to the *JCI* by Godoy-Molina et al. implying that elesclomol-copper (ES-Cu-Captisol) treatment improved neurodevelopmental outcomes, hair structure/pigmentation, and various neurochemical levels in two infants with Menkes disease caused by loss-of-function ATP7A variants. A critical factor not adequately explored is how the authors separated the effect of ES-Cu-Captisol on these parameters from that of copper histidinate (CuHis), an alternative copper formulation that each infant received concurrently (1). In fact, both infants (NP#1 and NP#2) received ES-Cu-Captisol only 1 day per week, versus CuHis for 6 days per week. Based on the respective doses reported, the percentage of weekly copper received from the ES-Cu-Captisol formulation was 7.7% of the total for NP#1 and 4.46% for NP#2. From scientific, medical, and clinical trial perspectives, it is problematic to “suggest that ES-Cu has therapeutic benefits on various tissues, particularly the brain” under these circumstances (1).

The enhanced survival and variably improved neurodevelopmental outcomes in response to early treatment for NP#1 and NP#2 are laudable in the face of this difficult illness and entirely similar to those reported in larger Menkes disease cohorts treated with CuHis alone (2–4), including subjects with complete loss-of-function *ATP7A* variants (4). Any clinical and biochemical benefits in individuals NP#1 and NP#2 are therefore impossible to attribute to ES-Cu-Captisol.

In contrast, the documented risks of ES-Cu-Captisol are notable. Godoy-Molina et al. report skin reactions to the drug (Supplemental Figure 2C) involving adipocyte necrosis in both individuals after subcutaneous injections of ES-Cu-Captisol (1). In the personal care and treatment of more than 150 patients with Menkes disease receiving CuHis injections (2–4), I have never witnessed skin reactions of this severity. The authors do not address the possible mechanism of this treatment-emergent adverse event, which may be related to the cellular toxicity and apoptotic effects of ES (5).

## Acknowledgments

Note added in proof: The U.S. Food and Drug Administration announced approval of copper histidinate for Menkes disease on January 12, 2026.

## Version 1 02/16/2026

Electronic publication
